# Role of Superoxide Reductase FA796 in Oxidative Stress Resistance in *Filifactor alocis*

**DOI:** 10.1038/s41598-020-65806-3

**Published:** 2020-06-08

**Authors:** Arunima Mishra, Ezinne Aja, Hansel M Fletcher

**Affiliations:** Division of Microbiology and Molecular Genetics, Department of Basic Sciences, School of Medicine, Loma Linda University, Loma Linda, California USA

**Keywords:** Biotechnology, Microbiology

## Abstract

*Filifactor alocis*, a Gram-positive anaerobic bacterium, is now a proposed diagnostic indicator of periodontal disease. Because the stress response of this bacterium to the oxidative environment of the periodontal pocket may impact its pathogenicity, an understanding of its oxidative stress resistance strategy is vital. Interrogation of the *F. alocis* genome identified the *HMPREF0389_00796* gene that encodes for a putative superoxide reductase (SOR) enzyme. SORs are non-heme, iron-containing enzymes that can catalyze the reduction of superoxide radicals to hydrogen peroxide and are important in the protection against oxidative stress. In this study, we have functionally characterized the putative SOR (FA796) from *F. alocis* ATCC 35896. The recombinant FA796 protein, which is predicted to be a homotetramer of the 1Fe-SOR class, can reduce superoxide radicals. *F. alocis* FLL141 (*∆FA796*::*ermF*) was significantly more sensitive to oxygen/air exposure compared to the parent strain. Sensitivity correlated with the level of intracellular superoxide radicals. Additionally, the *FA796*-defective mutant had increased sensitivity to hydrogen peroxide-induced stress, was inhibited in its ability to form biofilm and had reduced survival in epithelial cells. Collectively, these results suggest that the *F. alocis* SOR protein is a key enzymatic scavenger of superoxide radicals and protects the bacterium from oxidative stress conditions.

## Introduction

All living cells in an oxygen-rich environment encounter oxidative stress due to the generation of reactive oxygen species (ROS), including superoxide radicals, hydroxyl radicals and hydrogen peroxide (H_2_O_2_)^1,2^. Survival of organisms including bacteria must have systems that detoxify ROS and overcome their toxic outcomes due to the damage to cellular components including nucleic acids, membrane lipids and proteins^3,4^. An aerobic bacterial lifestyle will generally use superoxide dismutase (SOD) and catalase enzymes among others (including thioredoxin and peroxidase) as part of their defense against a ROS challenge^5–7^. SOD catalyzes the dismutation of superoxide radical into H_2_O_2_ and water^7^. Catalase converts H_2_O_2_ to oxygen and water^8^. This “canonical” defense strategy is less apparent or mostly absent in anaerobes that do not produce ROS through respiration but do require protection from exposure to oxygen molecules formed in other metabolic processes^9^. Anaerobes can exploit an alternative enzyme called superoxide reductase (SOR) to scavenge superoxide radicals^10^. SORs are referred to as a ‘novel paradigm’ for ROS detoxification^11^ and have been described as non-heme, iron-containing enzymes which catalyze the one electron reduction of superoxide radical to H_2_O_2_ in anaerobic organisms (Eq. 1)^10–13^.

1 $${{{\rm{O}}}_{2}}^{\cdot -}+{2{\rm{H}}}^{+}+{{\rm{e}}}^{-}\to {{\rm{H}}}_{2}{{\rm{O}}}_{2}$$

Unlike SODs, SORs, selectively reduce, rather than dismutate superoxide radical at an unusual [Fe(His)_4_Cys] catalytic site^13^. In the 1990s, the very first enzymes exhibiting SOR activity, unbeknownst to scientists at that time, were isolated from the sulfate reducing bacteria in the *Desulfovibrio* (*D*.) genus. These enzymes were desulfoferrodoxin (Dfx) from *D. vulgaris* Hildenborough and *D. desulfuricans* and later neelaredoxin (Nlr) from *D. gigas*^14,15^.

With their initial isolation from anaerobic bacteria and archaea, SORs are now present in all domains of life. They are evolutionarily diverse with their clustering based more on their amino acid sequence and less according to the phylogeny of the organisms^7^. Two general mechanistic groups of SORs [1Fe-SORs (Nlrs) and 2Fe-SORs (Dfxs)] have been described^12,13^. The catalytic [Fe(His)_4_Cys] site, a characteristic feature of SOR, is also known as the ‘SOR site’/‘Center II’ site. The 1Fe-SOR prototype, identified in *D. gigas* and *Pyrococcus furiosus*, is characterized as a homotetrameric, single-domain protein that carries the SOR site as the only prosthetic group^15,16^. The 2Fe-SOR prototype from *D. desulfuricans*, *D. vulgaris* and *Desulfoarculus baarsii* is homodimeric and contains an extra N-terminal domain with an [Fe(Cys)_4_] site, also referred as ‘Center I’^14,17^. In the 2Fe-SORs, the SOR site is present at the C-terminal domain and is structurally homologous to the 1Fe-SOR type^18^. SORs from the anaerobic spirochetes *Treponema pallidum* and *Treponema denticola* are categorized as Class III SORs^19–21^. They carry the two-domains and C-terminal SOR site, similar to the 2Fe-SORs. However, their N-terminal domains do not contain all 4 ‘Center I’ cysteine residues and also do not bind to any metal ion. Instead, they contain variable numbers (0, 1, 2, 3, 5, or 6) of cysteine residues^18^, for example, the N-terminal region of the *T. pallidum* SOR sequence contains only 1 cysteine residue, whereas the *T. denticola* SOR has 3 cysteine residues^19,21^.

*Filifactor alocis*, is a newly recognized, fastidious, Gram-positive, anaerobic periodontopathogen^22–24^. Periodontal disease is one of the most common inflammatory infectious diseases worldwide that has been linked to systemic diseases such as coronary heart disease^25^, rheumatoid arthritis^26^ and Alzheimer’s disease^27,28^. Associated with this disease is a shift in the host inflammatory response leading to destruction of soft and hard gingival tissues^29^. In comparison to the traditional periodontal pathogens (the “red complex” bacteria *Porphyromonas gingivalis*, *Tannerella forsythia* and *T. denticola*), *F. alocis* is seen in significantly more abundant numbers in diseased periodontal pockets, with undetectable levels in healthy or periodontitis-resistant patients^22–24,30^. Currently, little is known about the ability of *F. alocis* to combat the oxidative stress environment and persist in the periodontal pocket. This study is aimed to understand how *F. alocis* copes with exposure to oxygen and detoxifies the ROS in order to survive in the inflammatory environment of the periodontal pocket. Inspection of the *F. alocis* genome did not show the classical antioxidant enzymes catalase and SOD, however, it does encode a putative SOR (HMPREF0389_00796). We have overexpressed, purified and functionally characterized HMPREF0389_00796 (FA796) and established its expected *in vitro* SOR activity. More importantly, by modifying the gene manipulation methodology available for other periodontal pathogens, we have generated the first mutant (*∆FA796*) in *F. alocis* and show that *FA796* plays an important role in the ROS-detoxification pathway, biofilm formation and host cell survival in this bacterium.

## Results

### *F. alocis* ATCC 35896 is resistant to air exposure up to 2 hours

**Figure 1:**
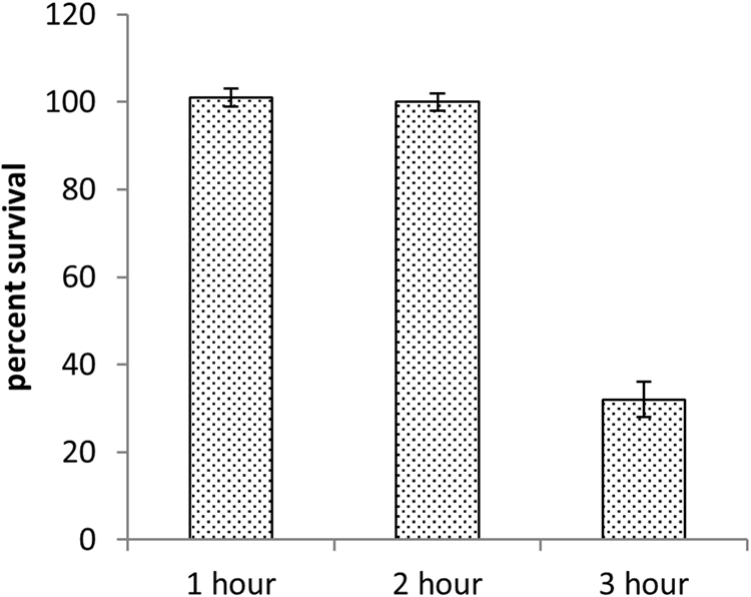
Air sensitivity of *F. alocis* wild-type ATCC 35896. *F. alocis*, on BHI agar plates (without cysteine), was exposed to air. At the indicated time periods, the subsets of plates were returned to the anaerobic chamber, incubated at 37 °C and colonies were counted after 5–7 days. CFU/mL was determined and percent survival was calculated as the ratio of CFU/ml of the air exposed bacteria versus the unexposed input. The results represent the means of three independent experiments. Error bars represent the standard deviations from the means. Wild-type *F. alocis* ATCC 35896 can tolerate atmospheric air exposure up to 2 h with a 100% survival rate.

*F. alocis* has to face constant oxidative stress (such as transient exposure to oxygen, superoxide radical and H_2_O_2_) in order to survive in the inflammatory environment of the periodontal pocket. To determine, whether the bacterium can tolerate some oxygen when exposed to air, wild-type *F. alocis* on BHI agar plates, was exposed to atmospheric air and its percent survival determined. As shown in Fig. 1, the wild-type *F. alocis* ATCC 35896 can tolerate atmospheric air exposure up to 2 hours (h) with a 100% survival rate.

### FA796 is homotetrameric and belongs to 1Fe-SOR class

**Figure 2:**
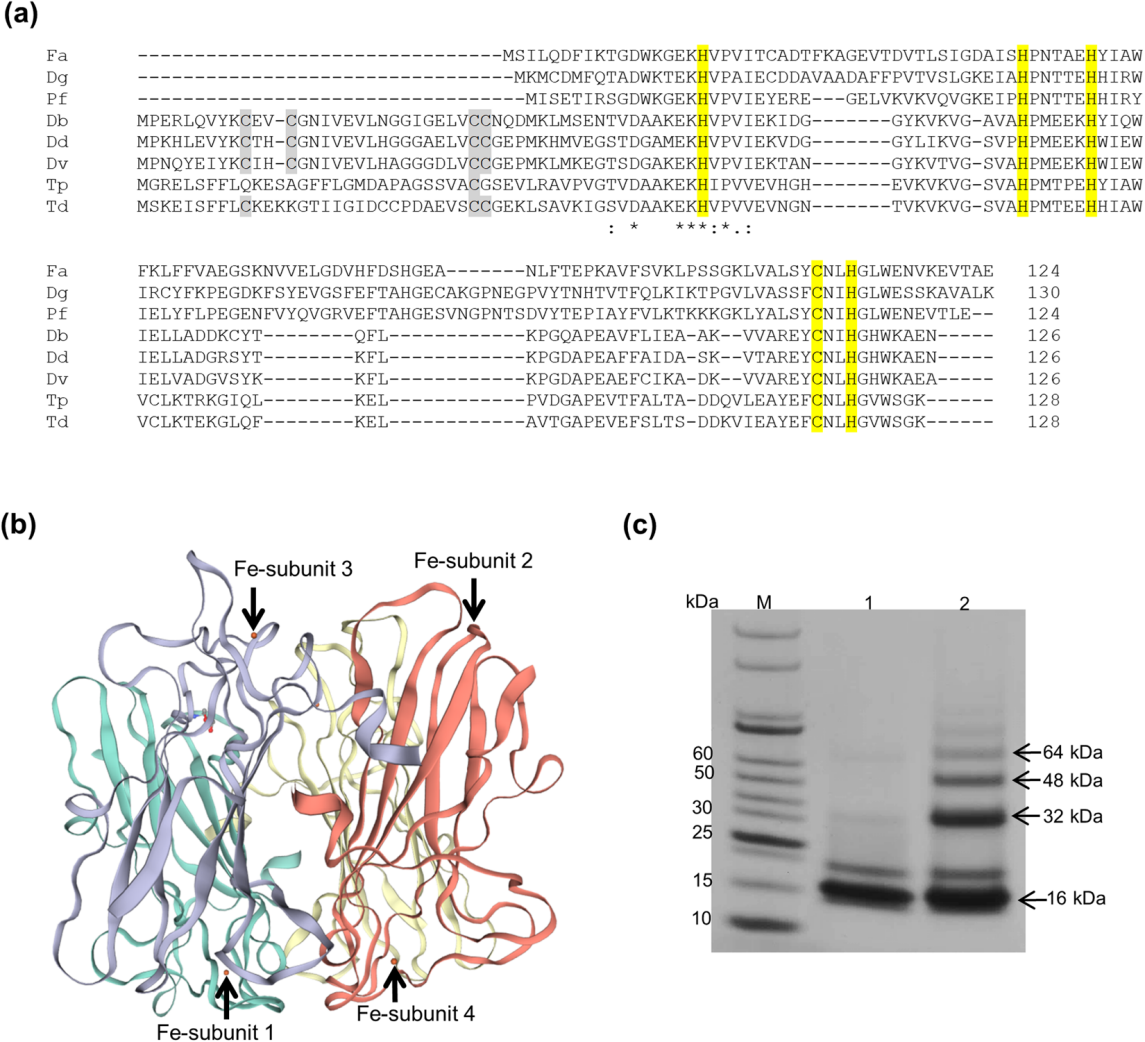
*F. alocis* SOR is homotetramer and belongs to 1Fe-SOR family. (**a**) Amino acid sequence alignment of *F. alocis* SOR (FA796) with other characterized SORs from different bacteria. From top to bottom are shown SORs from *F. alocis* (Fa), *D. gigas* (Dg), *P. furiosus* (Pf), [1Fe-SOR members]; *D. baarsii* (Db), *D. desulfuricans* (Dd), *D. vulgaris* Hildenborough (Dv), [2Fe-SOR members]; *T. pallidum* (Tp), and *T. denticola* (Td) [class III SORs]. Sequences were aligned using Clustal Omega version 2.0. The 4 histidine and 1 cysteine residues making the SOR site are highly conserved and highlighted in yellow. “Canonical” center I cysteine residues (Db, Dd and Dv SORs) and “noncanonical” cysteine residues (Tp and Td SORs) are grey-highlighted. (**b**) Ribbon diagram of the homotetrameric arrangement of *F. alocis* SOR. The model was generated by SWISS-MODEL using the *P. furiosus* SOR structure (PDB ID 1D06) as template. The entire homotetramer structure is shown with individual subunits (1–4) of the tetramer are colored in green, red, blue and yellow respectively. The four iron atoms in tetramer are shown as orange spheres. (**c**) 100 µM of r-FA796 was crosslinked with 0.1 mM disuccinimidyl suberate in 100 µl conjugation buffer (100 mM sodium phosphate pH 8.0) for 1 h at room temperature. High molecular weight complexes (32, 48 and 64 kDa corresponding to dimer, trimer and tetramer) are shown in lane 2 (lane 1: r-FA796 control and lane 2: FA796 crosslinked with disuccinimidyl suberate).

In order to identify the potential gene product which may protect *F. alocis* from oxidative stress, its genome was screened for typical antioxidant enzymes. The *F. alocis* genome does not encode open reading frames for any catalase or SOD. However, a 13.5 kDa protein encoded by the *HMPREF0389_00796* (*FA796*) gene and annotated as SOR was observed. SOR enzymes are known to protect anaerobes from air/oxygen exposure^21^. Most of the known SORs can be classified as either 1Fe- or 2Fe-SORs on basis of homology with other characterized SORs. To determine, if FA796 belongs to the 1Fe- or 2Fe-SOR class, its amino acid sequence was aligned with SORs from *D. gigas*, *P. furiosus* (1Fe-SOR members), *D. baarsii*, *D. desulfuricans*, *D. vulgaris* Hildenborough (2Fe-SOR members), *T. pallidum* and *T. denticola* (class III SORs). As shown in Fig. 2a, FA796 contains only 4 histidine and 1 cysteine (highlighted in yellow) residues [but not the cysteine residues (grey highlighted) of “Center I” of 2Fe-SORs] involved in the SOR active site. It shares 43% and 47% sequence identity with *D. gigas* and *P. furiosus* SOR respectively. The data taken together could suggest that FA796 belongs to the 1Fe-SOR class.

All the characterized 1Fe-SORs are shown to be homotetrameric. To confirm, if FA796 is also homotetrameric, a homology structural model of FA796 was generated using the automated mode of SWISS-MODEL server (Fig. 2b). Under those conditions, based on amino acid sequence homology, the server selected the *P. furiosus* homotetramer crystal structure (PDB ID 1D06) as template. FA796 is organized as a homotetramer composed of 4 subunits of 13.5 kDa with each subunit containing a single non-heme iron. The individual 1–4 subunits of the homotetramer are colored in green, red, blue and yellow respectively. The four iron molecules in tetramer are shown as orange spheres (Fig. 2b). Further, when FA796 was crosslinked using disuccinimidyl suberate, bands of different sizes (32, 48 and 64 kDa corresponding to dimer, trimer and tetramer) were observed on SDS-PAGE gel (Fig. 2c). Collectively, these results show that the *F. alocis* SOR encoded by *FA796* gene is homotetramer and would be consistent with the 1Fe-SOR class.

### UV-visible spectra of r-FA796

**Figure 3:**
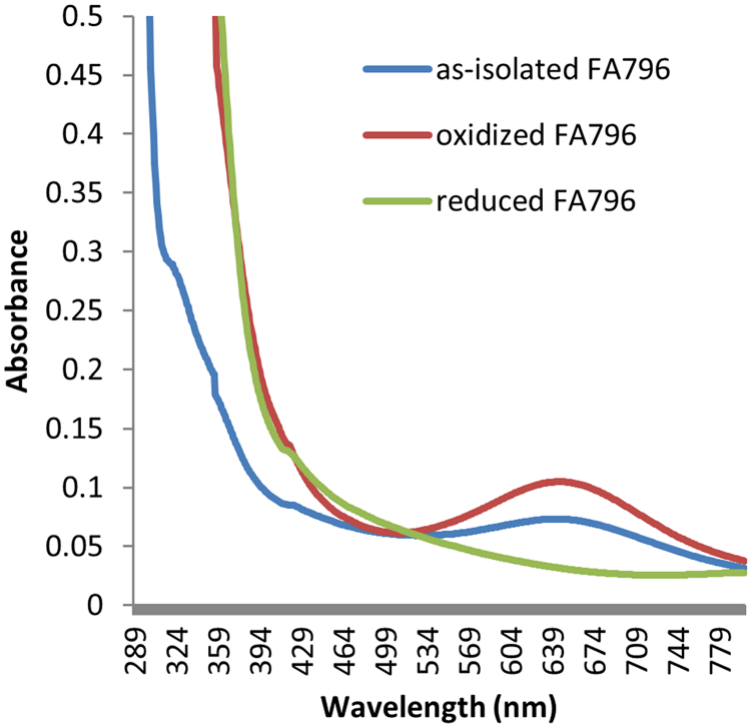
UV-visible spectra of r-FA796. A 50 µM protein solution (in 50 mM Tris.HCl, pH 7.5) was used to record the spectrum of as-isolated FA796 protein (as-isolated FA796). To get the spectrum of the oxidized protein, 50 µM as-isolated protein was treated with 75 µM of potassium ferricyanide (oxidized FA796). The oxidized protein sample was reduced by adding 75 µM β-mercaptoethanol (reduced FA796).

Figure 3 shows the UV-visible spectra of as-isolated, oxidized and reduced forms of the FA796 protein. The concentrated as-isolated r-FA796 protein was pale blue in color and showed a maximum absorbance peak at 644 nm (Fig. 3; as-isolated FA796 spectrum). When the protein was oxidized with potassium ferricyanide, the color of the protein changed to dark blue and the intensity of the band at 644 nm increased (Fig. 3; oxidized FA796 spectrum). Treatment of the oxidized protein with β-mercaptoethanol resulted in the disappearance of the blue color as well as loss of the absorption peak at 644 nm (Fig. 3; reduced FA796 spectrum). In the presence of DTT and dithionite similar UV-visible spectra were observed (data not shown). The blue color of the protein is consistent with the oxidized (ferric) form which is expected to show a broad peak in the visible region of the spectrum from 550 to 800 nm with maximum absorbance at 644 nm. Taken together, it is likely that the iron-center of the SOR site in the as-isolated r-FA796 protein exists as a mixture of both ferric and ferrous states^20^.

### Superoxide reductase activity of recombinant FA796 (r-FA796)

**Figure 4:**
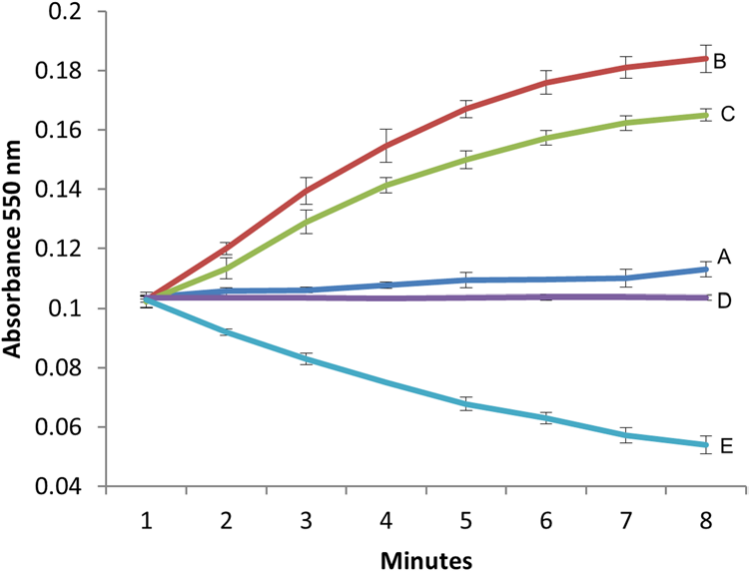
Superoxide reductase activity of FA796 in a cytochrome c assay. The assay was performed in 96-well plate under aerobic conditions, in a 100 µl total reaction volume in a buffer containing 100 mM Tris.HCl and 150 mM NaCl, pH 8.0. The reactions were followed by measuring absorbance at 550 nm for 10 minutes with 1 minute interval. (**A**) Cytochrome c (20 µM) was reduced in aerobic solutions resulting in an increased absorbance at 550 nm. (**B**) Generation of superoxide radicals by addition of xanthine (0.2 mM) and xanthine oxidase (0.0005 units) to the reaction mixture strongly enhanced the reduction of cytochrome c. (**C**) Addition of r-FA796 (1 µM) to the reaction mixture inhibited the reduction of cytochrome c by competing for the superoxide radicals. (**D**) Addition of 5.3 µM FA796 protein completely prevented the reduction of cytochrome c. (**E**) Addition of excess FA796 (10 µM) caused oxidation of the reduced cytochrome c. The results represent the means of three independent experiments. Error bars represent the standard deviations from the means.

To investigate if FA796 can catalyze the reduction of superoxide radicals, SOR activity of the r-FA796 protein was measured using the cytochrome c reduction assay (Fig. 4). Cytochrome c was reduced in aerobic solutions resulting in an increased absorption at 550 nm [Abs_550_] (Fig. 4; trace A). Addition of xanthine and xanthine oxidase to cytochrome c generated superoxide radicals due to which reduction of cytochrome c and therefore Abs_550_ was drastically increased (trace B). When the r-FA796 protein (1 µM) was added to the reaction mixture, it inhibited the reduction of cytochrome c by competing for the superoxide radicals (trace C). Addition of more protein (5.3 µM) completely abolished the reduction of cytochrome c (trace D). When FA796 was added in excess (10 µM), it was able to oxidize the reduced cytochrome c (trace E). These results suggest that FA796 protein has the ability to reduce superoxide radicals.

### Production of H_2_O_2_ by FA796 mediated SOR reaction

**Figure 5:**
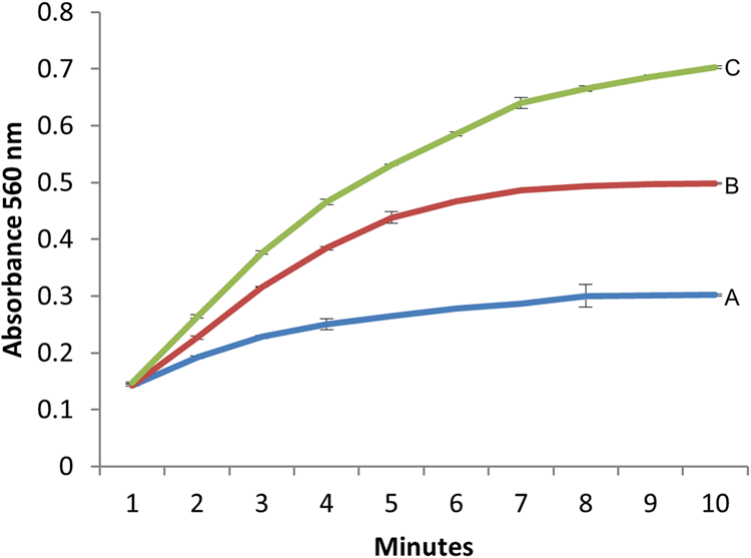
Measurement of H_2_O_2_ in the FA796 mediated SOR assay. Generation of H_2_O_2_ was determined by measuring the increase in absorbance at 560 nm due to the formation of resorufin. (**A**) H_2_O_2_ production by xanthine oxidase. No FA796 was added. (**B**) H_2_O_2_ production in the presence of 10 µM FA796 protein. (**C**) H_2_O_2_ production in the presence of 15 µM FA796 protein. The results represent the means of three independent experiments. Error bars represent the standard deviations from the means.

Production of H_2_O_2_ during cytochrome c reduction assay was measured by following the increase in absorbance at 560 nm due to the generation of resorufin. Resorufin was generated by the amplex red/horseradish peroxidase reaction in the presence of H_2_O_2_ (Fig. 5). Compared to control (no r-FA796; trace A), production of H_2_O_2_ was significantly increased in the presence of r-FA796 (traces B and C; 10 µM and 15 µM r-FA796 respectively).

### *FA796* contributes to survival of *F. alocis* exposed to air

**Figure 6:**
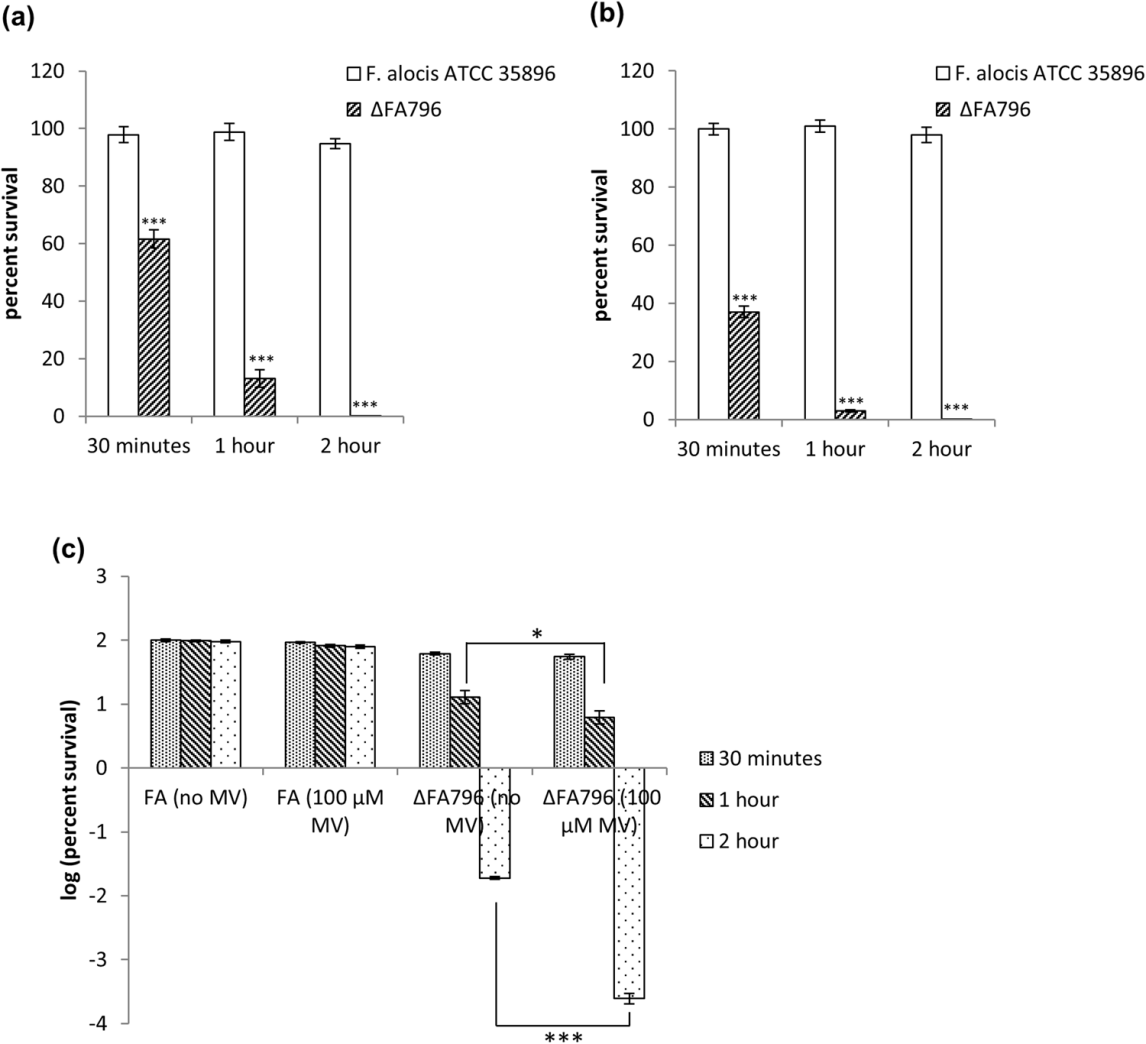
Survival of *F. alocis* wild-type and Δ*FA796* mutant exposed to air in (**a**) liquid cultures (**b**) on plates and (**c**) in liquid cultures in presence of methyl viologen (MV). (**a**) Strains grown in BHI broth (without cysteine) at 37 °C anaerobically were exposed to air. At the indicated time points, aliquots from air exposed cultures were transferred to the anaerobic chamber, serially diluted and plated on BHI agar plates. Colonies were counted after 5–7 days. (**b**) Strains on BHI agar plates (without cysteine) were exposed to air for the indicated time periods, the plates were then returned to the anaerobic chamber, incubated at 37 °C, and colonies were counted after 5–7 days. (**c**) Parallel sets of cultures containing either no MV or 100 μM MV were shaken while exposed to air at 37 °C. At the indicated time points, aliquots from each culture were transferred to the anaerobic chamber, serially diluted, plated on BHI agar plates and incubated at 37 °C. Colonies were counted after 5–7 days. For (**a**) and (**b**), CFU/mL were determined and percent survival was calculated. For (**c**), percent survival with and without MV was calculated for each culture, and the log values of percent survival were plotted. The results represent the means of three independent experiments. Error bars represent the standard deviations from the means. Statistical analysis was performed using two-tailed unpaired Student’s t-test (***p  <  0.005 and *p  <  0.5 vs. control).

In order to assess the contribution of *FA796* to the survival of *F. alocis* on exposure to air/oxygen, an isogenic mutant of *F. alocis* ATCC 35896 was constructed by replacing the *FA796* gene with an erythromycin antibiotic resistance cassette (*ermF*). After the electroporation of *F. alocis* with the purified fused PCR fragment (Materials and Methods), 4 colonies were detected on erythromycin plates after 5 days of incubation. The replacement of the *FA796* gene with the *ermF* cassette in these mutants was confirmed by PCR and DNA sequencing (data not shown). The isogenic mutant, designated FLL141 (Δ*FA796*::*ermF*) was selected for further studies. As shown in Fig. 6, the mutant is significantly more sensitive to aerobic stress compared to wild-type *F. alocis* strain. When grown in liquid cultures, following a 30 minute, 1 h and 2 h air exposure, there was a survival rate of 61%, 13% and 0.002% respectively, for the mutant strain (Fig. 6a). A more drastic difference in sensitivity was seen when the Δ*FA796* mutant, grown on BHI agar plates, was exposed to air (Fig. 6b). Compared to liquid cultures, the mutant had only 37%, 3% and 0.001% survival rates when exposed to air for 30 minutes, 1 h and 2 h respectively (Fig. 6b). In contrast, the wild-type *F. alocis* strain (grown either in liquid cultures or on BHI agar plates) did not show any change in sensitivity and had a 100% survival rate across all 3 time points (Fig. 6a, b). In the presence of methyl viologen which can produce intracellular superoxide radicals, the Δ*FA796* isogenic mutant showed increased sensitivity compared to the wild-type strain after 1 h and 2 h incubation periods (Fig. 6c). Altogether, the data suggest a role of *FA796* in protection of *F. alocis* during aerobic stress possibly through superoxide radical scavenging.

### H_2_O_2_-induced oxidative stress sensitivity of Δ***FA796*** mutant

**Figure 7:**
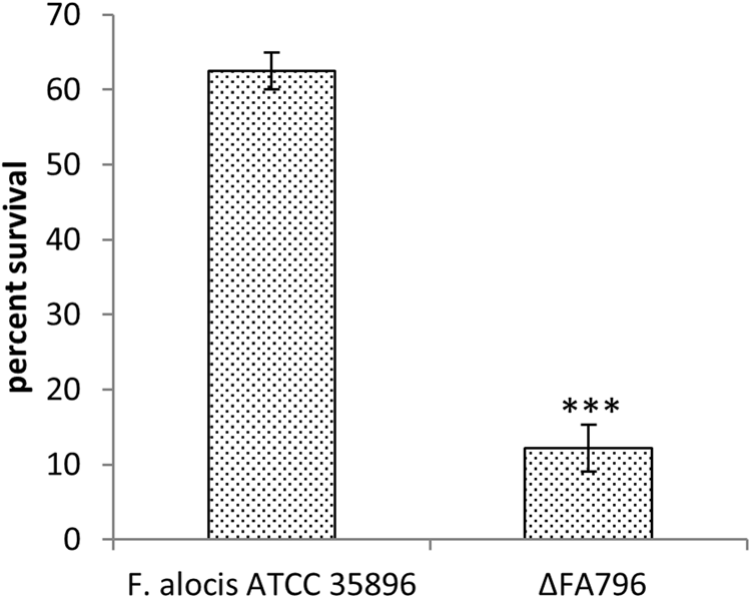
Survival of *F. alocis* wild-type and Δ*FA796* mutant exposed to H_2_O_2_. *F. alocis* strains were grown to early log phase (OD_600_ ~0.2) in BHI broth without cysteine. The cells were treated with H_2_O_2_ (0.25 mM) for 30 minutes, then plated on BHI agar plates and incubated in an anaerobic chamber at 37 °C for 5–7 days. The results presented here are the means of three independent biological experiments. Error bars represent the standard deviations from the means. Statistical analysis was performed using two-tailed unpaired Student’s t-test (***p  <  0.005 vs. control).

To ascertain, if *FA796* also plays a role in H_2_O_2_ (another source of oxidative stress in periodontal pocket) sensitivity, wild-type *F. alocis* and Δ*FA796* mutant were grown in the presence of H_2_O_2_ and their percent survival determined. As shown in Fig. 7, the *F. alocis* Δ*FA796* isogenic mutant had increased sensitivity to H_2_O_2_. Following a 30 minute exposure to 0.25 mM H_2_O_2_, the wild-type strain and Δ*FA796* mutant had 62.5% and 12.2% survival rates, respectively (Fig. 7).

### Δ***FA796*** mutant does not make biofilm compared to wild-type strain

**Figure 8:**
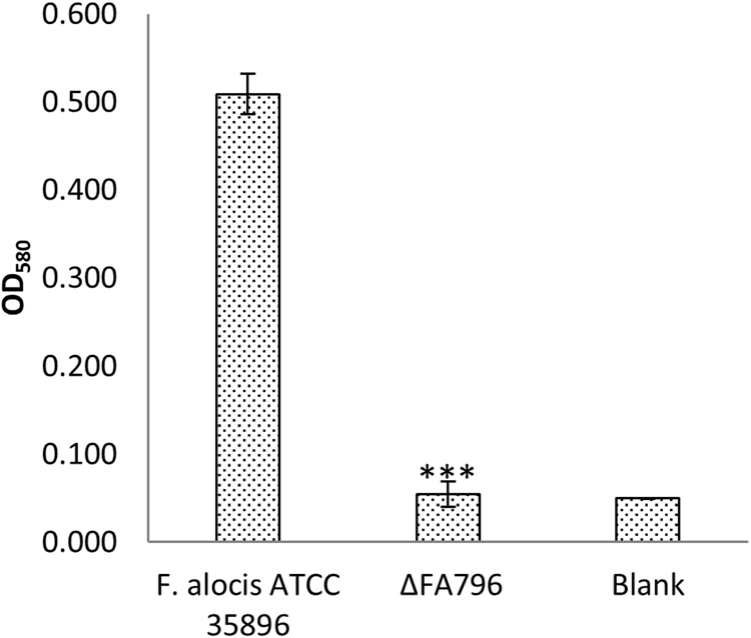
Role of *F. alocis* SOR in monospecies *in vitro* biofilm formation. *F. alocis* strains were grown in 96-well plates in BHI broth for 48 h. Bacterial biofilms were stained with 0.5% crystal violet and quantitated by measuring the absorbance at 580 nm. BHI broth was used as blank. The values presented here are the means of four independent experiments. Error bars represent the standard deviations from the means. Statistical analysis was performed using two-tailed unpaired Student’s t-test (***p  <  0.005 vs. control).

To test the potential role of FA796 in monospecies biofilm formation, both wild-type *F. alocis* and Δ*FA796* mutant strains were grown in 96-well plates and their ability to form *in vitro* biofilm was determined. As shown in Fig. 8, the biofilm formation capacity of the Δ*FA796* mutant was significantly reduced compared to the wild-type strain. These results show that FA796 can affect the *in vitro* formation of *F. alocis* biofilms.

### *FA796* plays a role in survival of *F. alocis* in host cells

**Figure 9:**
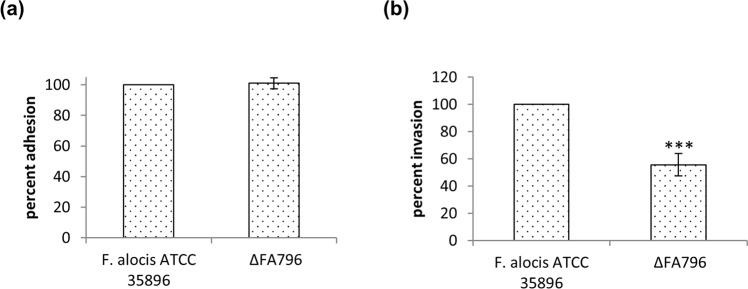
Adherence (**a**) and invasion (**b**) of *F. alocis* wild-type and *∆FA796* mutant in TIGK cells. Cells were grown and maintained at 37 °C under 5% CO_2_ in DermaLife K Serum‐Free Keratinocyte Culture Medium with supplements. Epithelial cells (10^5^ cells) were infected with 10^7^ bacteria (MOI:100) for 1 h in an anaerobic chamber. For the invasion assay, extracellular bacteria were killed by incubating the cells with 200 μg/ml of metronidazole for 1 h. The percent adhesion and invasion were calculated and data are presented as percentage to that of wild-type, which was set to 100%. Experiments were carried out in three independent repeats in triplicates. Error bars represent the standard deviations from the means. Statistical analysis was performed using two-tailed unpaired Student’s t-test (***p  <  0.005 vs. control). The mutant did not show any change in adhesion (**a**), however its invasion ability was reduced to 55% (**b**) compared to the wild-type *F. alocis* strain.

Adherence and invasion of periodontopathogens into periodontal tissues is an important step that can initiate periodontal diseases. Gingival epithelial cells are among the first host cells encountered by periodontal bacteria. Therefore, the adhesion and invasion abilities of *F. alocis* wild-type and *∆FA796* mutant in telomerase-immortalized gingival keratinocytes (TIGKs) were determined. As shown in Fig. 9, adhesion of *∆FA796* mutant was similar to wild-type *F. alocis* (Fig. 9a). In contrast, the mutant exhibited a significant reduction in the ability to invade the TIGK cells compared to the wild-type strain. Following 1 h incubation, the *∆FA796* mutant had only 55% survival rate compared to the wild-type strain which was set to 100% (Fig. 9b). These results suggest that *FA796* may play a role in survival of *F. alocis* in the host cells.

## Discussion

Oxidative stress has been shown to be involved in the progression of periodontal disease which is a complex polymicrobial chronic inflammatory condition involving destruction of the tissues supporting the teeth^29,31,32^. In the USA, over 65 million people are affected by periodontitis and approximately 50% of the adults in the country struggle with one or more forms of this disease^33,34^. The tissue destruction and concomitant induction of oxidative stress can arise from an excessive inflammatory response to bacterial plaque with the accompanying release of ROS, such as H_2_O_2_ and superoxide radicals from neutrophils^35–38^. Recent microbiome studies and advanced sequencing have facilitated the identification of several novel emerging oral pathogens such as *F. alocis*, which show strong correlation with the disease^22–24^. Because of the chronic inflammation and other prevailing conditions including fluctuations in nutrient availability, pH, oxygen tension, and the presence of other microbial species, *F. alocis* must have properties that will allow it to colonize and survive in the stress environment of the periodontal pocket. Some of the recent studies have shown that *F. alocis* is comparatively more resistant to H_2_O_2_-induced oxidative stress, can form biofilm and also subvert the host defense system^23,39^. In addition, it has the ability for prolonged survival (up to 4 h) inside neutrophils^40^. These observations suggest that *F. alocis* may likely have the inherent ability to detoxify the oxidative stress environment of the periodontal pocket. Currently, the genes that may play a role in oxidative stress resistance mechanism(s) in *F. alocis* are unknown or implied at best. In this study, we have functionally characterized the first gene in *F. alocis*, which encodes for an antioxidant enzyme SOR. SORs are iron-containing enzymes that effectively catalyze the reduction of superoxide radicals to H_2_O_2_ [with electrons from NAD(P)H] which is further metabolized to water, thus forming a defense mechanism^7,10–13^. Based on the primary amino acid sequence and predicted model, the *F. alocis* SOR likely belongs to the 1Fe-SOR class (Fig. 2). Our *in vitro* assay using the r-FA796 protein confirmed that FA796 can reduce superoxide radicals to H_2_O_2_ (Figs. 4 and 5). The reaction catalyzed by SOR generally requires at least one electron donor (transferring the electron to SOR) and one oxidoreductase (which generates the reduced form of electron donor for the next cycle). Mostly, the small electron transfer protein, rubredoxin, has been proposed to be the proximal electron donor to both 1Fe- and 2Fe-SORs^41^. *F. alocis* carries genes that encode proteins HMPREF0389_00337 and HMPREF0389_RS04690 annotated as rubredoxin and ‘rubredoxin like protein’ respectively. Though we did not find any NADH: rubredoxin oxidoreductase (NROR) in the genome, it does have a gene HMPREF0389_00379 which encodes NAD(P)/FAD-dependent oxidoreductase. Whether *F. alocis* SOR reduces superoxide radicals to H_2_O_2_ involving FA337 and/or FA_RS04690 and oxidoreductase (FA379) is not confirmed and is the subject of further investigation in the laboratory.

One intriguing question is the fate of the SOR reaction product H_2_O_2_ which is as detrimental as the parent superoxide. Indeed, *F. alocis* is sensitive to H_2_O_2_ (Fig. 7). In most organisms, H_2_O_2_ is detoxified by catalases and peroxidases. Some of the other H_2_O_2_ scavenging enzymes in anaerobes include bacterioferritin comigratory protein (Bcp), ruberythrin and alkyl hydroperoxide reductase enzyme system (AhpC/AhpF)^8,42^*. F. alocis*, lacks genes coding for catalases and ruberythrin (https://www.ncbi.nlm.nih.gov/nuccore/NC_016630.1). However, it does encode for a sole peroxidase in the genome, glutathione peroxidase (HMPREF0389_01233). Additionally, the product of *F. alocis* gene *HMPREF0389_00768* is annotated as alkyl hydroperoxide reductase subunit AhpC, though its partner AhpF is missing in the genome. When the *F. alocis* genome was interrogated with *P. gingivalis* W83 AhpF (PG0619) as query, HMPREF0389_00608 (annotated as thioredoxin-disulfide reductase) showed 34% identity. Interestingly, *F. alocis* AhpC (HMPREF0389_00768) also showed 30% identity with *P. gingivalis* W83 Bcp (PG0880). A potential mechanism by which *F. alocis* eliminates H_2_O_2_ may involve either one or all of these enzymes and should be confirmed with further experiments.

Due to the difficult genetic manipulation of many anaerobes, very few *in vivo* studies involving SOR mutants are available. Likewise, molecular genetics studies in *Filifactor* have been hampered due to the lack of an efficient genetic system to allow genetic manipulations of the genome. To date, none of the *F. alocis* genes have been fully functionally characterized. In this study, we have constructed the first gene deletion mutant (*∆FA796*) in *F. alocis*. The *∆FA796* mutant was sensitive to air after 30 minutes of exposure and it was not viable after 2 h (Fig. 6). These results are similar to the *T. denticola* SOR mutant that was previously shown to be significantly more sensitive to atmospheric air^21^. However, in contrast to *T. denticola, F. alocis* was relatively more resistant to air exposure for up to 2 h (Fig. 6). Although it has been suggested that the stress responses to superoxide radical and H_2_O_2_ (product of SOR reaction) are distinct^4^, the *∆FA796* mutant in our study was significantly more sensitive to H_2_O_2_ (Fig. 7). Taken together, this may raise the possibility that the stress responses to superoxide radical and H_2_O_2_ in *F. alocis* may share a similar/common regulatory pathway(s). To our knowledge, there are no reports of H_2_O_2_ sensitivity in other bacterial SOR mutant strains. However, the Δ*sodA* mutant (H_2_O_2_ is also product of SOD reaction) in *Streptococcus suis* was shown to be more susceptible to oxidative stresses induced by both H_2_O_2_ and superoxide radicals^43^. In contrast, a Δ*sod* mutant in *P. gingivalis* was only sensitive to air but not to H_2_O_2_^44^.

The prevalence of *F. alocis* in the periodontal pocket is likely due in part to its ability to form biofilm which is known to be a primary initiating factor of bacterial-induced periodontal disease^23^. Moreover, bacterial cells in biofilms are physiologically different from planktonic cells, often exhibiting increased resistance to environmental stress^45^. For example, gene expression of *P. gingivalis* ATCC 33277 grown in an *in vitro* multispecies biofilm revealed the up-regulation of ROS detoxification and peroxides removal systems^46^. This is consistent with other observations that demonstrate the modulation of biofilm formation by oxidative stress in bacteria^47^. The accumulation of ROS can enhance or inhibit biofilm formation^48,49^. The results from this study confirm a role for the *F. alocis* SOR in biofilm formation (Fig. 8). Because the *F. alocis ∆FA796* mutant showed a decreased ability to form biofilm suggest that accumulation of ROS in this mutant may have an inhibitory effect on this phenotype.

*F. alocis* has virulence attributes that include the invasion of epithelial cells^39,50^. Our data have provided evidence that the *F. alocis* SOR mutant did not impair adhesion of the bacterium but reduced invasion and had a significant effect on its ability to survive in host cells compared to the wild-type (Fig. 9). The production of ROS in epithelial cells, the first barrier met by pathogens, can be triggered as a response to infection and plays a role in innate host defense^51^. There are also other reports of the production of ROS in nonphagocytic cells triggered by bacterial infections^52,53^. To promote survival, a mechanism to modulate this response has been observed in bacteria. In epithelial cells, infected with *Chlamydia trachomatis*, the production of ROS is turned on but rapidly shut down via the sequestering of a vital component for NADPH oxidase activity thus impairing one of the signaling pathways triggered by infection^51^. Because the *F. alocis* SOR (FA796) protein is a key enzymatic scavenger of superoxide radicals and can protect the bacterium from oxidative stress conditions, it is possible that the FA796-mediated attenuation of ROS may likely be a mechanism promoting the survival of *F. alocis* within the host cells. Further investigations are required to clarify this mechanism.

In summary, *F. alocis* SOR plays a vital role in defense against oxidative stress, affects biofilm formation and also helps the bacterium to survive in the host cells. Furthermore, the genetic manipulation methodology described here will help to decode pathogenic mechanisms of *F. alocis* in near future and will shed light on the overall role of this bacterium in the etiology of periodontal disease. As *FA796* is the first functionally characterized gene in *F. alocis*, this work delivers novel insights regarding the survival mechanisms of newly recognized *F. alocis* in the periodontal pocket and may reveal new therapeutic targets for disease treatment and prophylaxis that will have a positive impact on human health.

## Materials and Methods

### Bacterial strains, plasmids and growth conditions

Bacterial strains and plasmids used in this study are listed in Table 1. The *F. alocis* strains were grown in Brain Heart Infusion (BHI) broth supplemented with yeast extract (0.5%), hemin (5 µg/ml), vitamin K (0.5 µg/ml), cysteine and L-arginine (100 µM). *Escherichia coli* strains were grown aerobically in Luria-Bertani (LB) broth with shaking. Unless otherwise stated, all cultures were incubated at 37 °C*. F. alocis* strains were cultured in an anaerobic chamber (Coy Manufacturing) in 10% H_2_, 10% CO_2_ and 80% N_2_. The growth rates for *E. coli* and *F. alocis* strains were determined spectrophotometrically by measuring optical density at 600 nm (OD_600_). Antibiotics were used at the following concentrations: erythromycin 5 µg/ml (for *F. alocis*) and ampicillin 100 µg/ml (for *E. coli*).

**Table 1:** Bacterial strains and plasmids used in this study.

Strain or plasmid	Genotype and description	Reference or source
***F. alocis*** **strains**		
FA ATCC 35896	Wild-type strain	^60^
FLL141	Δ*FA796*::*ermF,* an isogenic derivative of FA ATCC 35896	This study
***E. coli*** **strains**		
Top10	Used for general cloning purpose	Invitrogen
BL21 DE3	F^–^ *omp*T *hsd*S_B_ (r_B_^–^, m_B_^–^) *gal dcm rne*131 (DE3), used as protein expression strain	Invitrogen
**Plasmids**		
pVA2198	Sp^r^, *ermF-ermAM*	^55^
pET21b	Ap^r^, C-terminal His-tag	Novagen
pET21b-*FA796*	Ap^r^, pET21b derivative expressing *FA796*	This study

### Construction of *F. alocis* FLL141 (∆*FA796*) mutant

The construction of an isogenic *FA796* mutant in *F. alocis* ATCC 35896 was done using a long PCR based fusion method as previously reported with some modifications^54^. Briefly, around 1 kb up- and 1 kb downstream fragments of *FA796* were PCR amplified from chromosomal DNA of *F. alocis*. The promoterless *ermF* cassette without a transcriptional terminator was amplified from the pVA2198 plasmid^55^ with primers containing overlapping bases for the up- and downstream *FA796* fragments respectively. Finally, the upstream fragment, *ermF* and downstream fragments were fused together and the purified fusion product was electroporated into *F. alocis* competent cells. The cells were plated on BHI agar plates containing 5 µg/ml of erythromycin and incubated at 37 °C for 5–7 days inside the anaerobic chamber. Colonies grown on erythromycin plates were screened for the correct gene replacement by PCR and DNA sequencing. Primers used in this study are listed in Supplementary Table 1.

### Electroporation of *F. alocis*

1 ml of an actively growing culture of *F. alocis* was used to inoculate 4 ml of BHI broth and incubated at 37 °C in the anaerobic chamber. Next morning, the cells from 5 ml culture (OD_600_ = 0.24) were harvested by centrifugation at 2,600 *g* for 10 min at 4 °C, washed and resuspended in 1 ml of ice cold electroporation buffer (10% glycerol, 1 mM MgCl_2_), and incubated on ice for 20 minutes. Cells were centrifuged again; the pellet was washed with 1 ml of 10% glycerol and finally resuspended in 100 µl of 10% glycerol. 1–4 µg of DNA was added to the 100 µl competent cells. The cell suspension was placed in a sterile electrode cuvette (0.2-cm gap) and then incubated on ice for 10 minutes. The cells were pulsed with a Bio-Rad gene pulser at 2.5 volts and 600 ohms. The cuvetts were immediately placed inside the anaerobic chamber, 500 µl of BHI broth was added to the *F. alocis* cells and incubated at 37 °C for approximately 24 h. Cells were then plated on BHI agar plates containing 5 µg/ml erythromycin and incubated anaerobically at 37 °C for 5 to 7 days.

### Cloning, expression and purification of r-FA796

The gene encoding FA796 was PCR amplified using *F. alocis* ATCC 35896 genomic DNA and a pair of oligonucleotide primers (Fa796-SacI-for and Fa796-HindIII-rev, Supplementary Table 1). The PCR product was then purified, digested with *Sac*I and *Hind*III and subsequently ligated into the plasmid pET21b which had been precut with *Sac*I and *Hind*III restriction enzymes. The resulting ligation mix was transformed into the *E. coli* TOP10 and plated on LB agar plates containing 100 µg/ml of ampicillin. Bacterial colonies were screened by colony PCR to identify the  positive clone, which was further verified by restriction enzyme digestion and DNA sequencing. The r-plasmid pET21b-*FA796* was then transformed into *E. coli* BL21 DE3 cells for expression.

A 1 liter culture of *E. coli* BL21 cells harboring the r-plasmid was grown at 37 °C in LB medium containing 100 μg/ml of ampicillin until OD_600_ ~0.6. FA796 expression was induced with 1 mM IPTG at 37 °C for 4 h. Once expressed, the protein was purified under native conditions using Ni-NTA resin according to the manufacturer’s instructions (Qiagen). The pure protein fractions were subsequently dialyzed twice against 1 liter of dialysis buffer containing 150 mM NaCl, 50 mM Tris.HCl, pH 7.5 at 4 °C and stored at −80 °C in 10% glycerol in aliquots.

### Biofilm formation assay

*In vitro* biofilm assay was done as previously described with minor modifications^56^. Briefly, *F. alocis* strains were grown overnight in BHI broth at 37 °C (OD_600_ ~0.22–0.24). Cultures were diluted to OD_600_ ~0.05 in BHI, and 200 µl of diluted culture was used to inoculate sterile, 96-well polystyrene plates (Greiner Bio-One) at 37 °C inside the anaerobic chamber. After 48 h, wells containing the stationary-phase cells with similar OD_600_ were used to perform biofilm assay. Free floating cells were aspirated and the attached biofilm growth of bacteria was gently washed three times with 200 µl of PBS and subsequently stained with 100 µl of 0.5% crystal violet for 30 minutes. The unbound dye was completely removed by washing several times with PBS. For quantitative analysis of biofilm production, biofilms in wells were destained with 100 µl of ethanol/acetone mix (80:20) for 15 minutes. Optical density at 580 nm was measured using xMark microplate spectrophotometer (Bio-Rad). BHI broth was used as blank. The assay was done in quadruplicates in four independent experiments.

### UV-visible spectroscopy

UV-visible absorption spectra of as-isolated, oxidized and reduced r-FA796 protein were obtained on Beckman DU640 spectrophotometer. FA796 protein was oxidized by treatment of 50 µM purified protein with 75 µM of potassium ferricyanide for 10 minutes at room temperature. Spectrum of the reduced protein was recorded after the oxidized protein was reduced with 75 µM of β-mercaptoethanol.

### Superoxide reductase activity assay

The ability of r-FA796 to catalyze the reduction of superoxide radical was determined by monitoring the inhibition of cytochrome c reduction by superoxide radicals generated from xanthine/xanthine oxidase reaction^10^. The assay was performed at room temperature in 96-well plate under aerobic conditions. Each reaction was carried out in a 100 µl total reaction volume containing a buffer (100 mM Tris.HCl and 150 mM NaCl, pH 8.0), 20 µM equine heart cytochrome c (Sigma), 0.2 mM xanthine (Sigma), 0.0005 units of xanthine oxidase (Sigma) and different concentrations of r-FA796. The reactions were followed by measuring absorbance at 550 nm for 10 minutes with 1 minute interval. Experiments were done in triplicate in three independent repeats.

### H_2_O_2_ measurement

FA796 mediated generation of H_2_O_2_ during cytochrome c assay was measured by using Amplex Red Hydrogen Peroxide/Peroxidase assay kit (Fisher) as per the manufacturer’s instructions. Briefly, 50 ul of a stock solution of Amplex Red (50 µM) and horseradish peroxidase (0.01 units) in 50 mM sodium phosphate buffer, pH 7.4, was added to the 50 µl of the sample mix containing xanthine, cytochrome c and r-FA796 (as required). Reactions were started by adding xanthine oxidase which generated superoxide radicals. In the presence of horseradish peroxidase, the amplex red reagent reacts with H_2_O_2_ and produces resorufin. Production of resorufin was followed by the increase in absorbance at 560 nm for 10 minutes.

### Air sensitivity of wild-type *F. alocis* and ∆*FA796* mutant

Air sensitivity of *F. alocis* strains was assessed by exposing either agar-plated bacterial cultures or liquid cultures to air [∼20% (v/v) oxygen]. For air exposure of bacteria on plates, liquid cultures of *F. alocis* strains [grown overnight (OD_600_ ∼0.24) in BHI broth, in the anaerobic chamber] were serially diluted and appropriate dilutions were plated on BHI agar plates. The plates (except for time point zero/input) were removed from the anaerobic chamber and placed outside in air at 37 °C. At designated time points (30 minutes, 1 h and 2 h), subsets of the plates were returned to the anaerobic chamber and incubated at 37 °C.

For liquid culture air exposure, overnight grown cultures of *F. alocis* (OD_600_ ∼0.23) in the anaerobic chamber, were shifted out of the chamber and incubated at 37 °C in an aerobic incubator shaking at 180 rpm. At set time points, aliquots of cells from outside aerobic incubator were transferred back to the anaerobic chamber, serially diluted and plated on BHI agar plates.

In order to study the effect of methyl viologen on air-exposed cultures, 10 ml cultures were grown overnight (OD_600_ of ∼0.23) in the anaerobic chamber. After removing an aliquot for time zero, the cultures were split into 2 parts (5 ml each). Methyl viologen (100 μM) was added to one part and the cultures were removed from the anaerobic chamber and shaken at 180 rpm in an aerobic incubator at 37 °C. Aliquots of each culture (with and without methyl viologen) were removed at 30 minutes, 1 h and 2 h intervals, moved back to the anaerobic chamber, serially diluted, and plated on BHI agar plates. Colonies were counted and CFU/mL was determined after 5–7 days of incubation at 37 °C. Percent survival was calculated as the ratio of CFU/ml of the air exposed bacteria versus the unexposed input. BHI broth and BHI agar plates used in above experiments did not contain any cysteine. Each experiment was done in triplicates, and repeated at least three times independently.

### H_2_O_2_ sensitivity assay

Sensitivity of *F. alocis* strains to H_2_O_2_ was tested as previously reported with some modifications^57^. Briefly, bacterial cultures were grown overnight in an anaerobic chamber in BHI broth (no cysteine) at 37 °C (OD_600_ ∼0.22). 5 ml of bacterial culture was subsequently treated with 0.25 mM H_2_O_2_ and incubated at 37 °C. After 30 minutes, H_2_O_2_ exposed bacterial cultures were serially diluted and appropriate dilutions were plated on BHI agar plates. Colonies were counted and CFU/ml was determined after 5–7 days incubation at 37 °C. Percent survival was calculated as the ratio of CFU/ml of H_2_O_2_ treated bacteria versus the untreated input. Experiments were done in triplicate in three independent repeats.

### Disuccinimidyl suberate crosslinking assay of FA796

For the crosslinking assay, 100 µM of r-FA796 was mixed with disuccinimidyl suberate (0.1 mM) in 100 µl conjugation buffer (100 mM sodium phosphate pH 8.0). The reaction mixture was incubated for 1 h at room temperature. Reaction was stopped by adding 50 mM Tris.HCl. 10 µl of the reaction mixture was analyzed on SDS-PAGE gel.

### Bioinformatics analysis

Nucleotide sequence of *FA796* gene (from *F. alocis* ATCC 35896) and protein sequences of different SORs were retrieved from the NCBI database. SORs were aligned using Clustal Omega version 2.0 (https://www.ebi.ac.uk/Tools/msa/clustalo/)^58^. Homology structure model of FA796 was generated using the SWISS-MODEL server (https://swissmodel.expasy.org/)^59^.

### Epithelial cell culture

TIGK cells were cultured in DermaLife K Serum‐Free Keratinocyte Culture Medium (Lifeline Cell Technology) supplemented with 0.5 ng/ml TGFα, 5 μg/ml insulin, 1 μM epinephrine, 5 μg/ml apo-transferrin, 100 ng/ml hydrocortisone, 0.4% bovine pituitary extract, and 6 mM L-Glutamine at 37 °C under 5% CO_2_.

### Cell adhesion and invasion assays

The adhesion and invasion assays were performed as described previously with minor modifications^60^. Briefly, overnight grown *F. alocis* cultures (OD_600_ ~0.23) were used to infect TIGK cells (grown in 12-well plates [1.0 × 10^5^ cells per well]) with a multiplicity of infection (MOI) of 100 (~10^7^ CFU/ml) for 1 h at 37 °C. After 1 h incubation, nonadherent bacteria were removed by washing the cells with PBS, washed cells were detached and lysed by Trypsin and 0.025% Triton X-100. The appropriate dilutions were plated on BHI agar plates to enumerate the adherent bacteria. For the invasion assay, following 1 h infection, the extracellular bacteria were killed by incubating the cells with 200 μg/ml of metronidazole for 1 h. The cells were then washed, detached and lysed. Lysates were serially diluted and plated on BHI agar plates. Plates from both adhesion and invasion assays were incubated anaerobically at 37 °C for 5–7 days after which the colonies were counted and CFU/ml was calculated. The CFU of adherent and/or invaded bacteria for each strain was compared with input titre and the percentage of adherent and invaded bacteria were determined. Each assay was performed in triplicates and repeated three times. As the ∆*FA796* mutant is sensitive to air, all the steps of this assay were done inside the anaerobic chamber.

## Supplementary information

Supplementary Information.

## Data Availability

All data generated or analyzed during this study are included in this article. Strain(s) generated during the study will be available from the corresponding author on request.
