# Supplementary material for: Role of Superoxide Reductase FA796 in Oxidative Stress Resistance in Filifactor alocis
Source: Sci Rep. 2020 Jun 8;10:9178. doi: 10.1038/s41598-020-65806-3 (PMC7280497; doi:10.1038/s41598-020-65806-3)
Supplement: Supplementary file 1 — Supplementary Information. [file 41598_2020_65806_MOESM1_ESM.pdf]

Role of Superoxide Reductase FA796 in Oxidative Stress Resistance in *Filifactor alocis*

Arunima Mishra, Ezinne Aja and Hansel M Fletcher

Supplementary Table 1. Primers used in this study

Primer	Sequence (5′-3′) [a, b]
P1-Fa796-up-for	CGACAAATATACAACTATTGAC
P2-Fa796-erm-rev	ATTTATTCCTCCTAGTTAGTCAAATATCCCCCTTATAATAAC
P3-erm-for	TGACTAACTAGGAGGAATAAATGACAAAAAAGAAATTGCCCG
P4-erm-rev	GATTATTCCCTCCAGGTACTACGAAGGATGAAATTTTTCA
P5-Fa796-erm-for	GTAGTACCTGGAGGGAATAATCAGGCATATAGGGCATTTGTAC
P6-Fa796-dn-rev	TAAAGTATTTACAGTAGGCGG
Fa796-SacI-for	AAA***GAGCTC***ATGAGTATTTTACAAGAT
Fa796-HindIII-rev	GAA***AAGCTT*** TTCAGCTGTCACTTCTTTC

[a] Complementary bases are underlined.

[b] Restriction sites in the primers are bold and italicized.
